# Validation of the virtual reality functional capacity assessment tool (VRFCAT) in a Spanish sample of individuals with psychosis and healthy controls

**DOI:** 10.1192/j.eurpsy.2025.10134

**Published:** 2025-11-18

**Authors:** César González-Blanch, Raquel López-Carrilero, Teresa Bobes-Bascarán, Ana Catalán, Claudia Aymerich, Roberto Rodriguez-Jimenez, Alfonso Gutiérrez-Zotes, Olimpia Díaz-Mandado, Ana González-Pinto, Guillermo Cano-Escalera, Marta Zubia, Daniel Guinart, Manuel del-Castillo-Serrano, Elisabet Vilella, Leticia García-Álvarez, Susana Ochoa, Patricia Correa-Ghisays, Vicent Balanzá-Martínez, Joan Vicent Sánchez-Ortí, Esther Pousa, Salvador Miret, Cristina Falip, Pablo Reguera-Pozuelo, Rafael Fernández-Martínez, Ignacio García-Cabeza, Carlos Campos-Rodriguez, Josep Maria Crosas, Jesus Cobo, Helena Pardina-Torner, Salvador Perona-Garcelán, Manuel Muñoz-Caracuel, Ángel Yorca-Ruiz, Víctor Ortiz-García de la Foz, Rosa Ayesa-Arriola

**Affiliations:** 1 Mental Health Centre, University Hospital Marqués de Valdecilla, Santander, Spain; 2 IDIVAL, Valdecilla Biomedical Research Institute, Santander, Spain; 3 Department of Psychology, International University of La Rioja (UNIR), Logroño, Spain; 4 Research Unit, Parc Sanitari Sant Joan de Déu, Sant Boi de Llobregat, Spain; 5 MERITT Research Group, Institut de Recerca Sant Joan de Déu, Esplugues de Llobregat, Spain; 6 Centro de Investigación Biomédica en Red de Salud Mental (CIBERSAM), Instituto de Salud Carlos III (ISCIII), Madrid, Spain; 7 Department of Psychology, University of Oviedo, Oviedo, Spain; 8 Department of Psychiatry, Servicio de Salud del Principado de Asturias (SESPA), Oviedo, Spain; 9 Psychiatric Research Group, Instituto de Investigación Sanitaria del Principado de Asturias (ISPA), Oviedo, Spain; 10 Behavioral and Mental Disorders Neuroscience Unit, INEUROPA, Oviedo, Spain; 11 Department of Neuroscience, University of the Basque Country (UPV/EHU), Leioa, Spain; 12 Department of Psychiatry, Basurto University Hospital, OSI Bilbao-Basurto, Bilbao, Spain; 13 Early Psychosis: Intervention and Clinical-Detection (EPIC) Lab, Department of Psychosis Studies, King’s College London, London, UK; 14 Psychiatry Service, Biobizkaia Health Research Institute, Barakaldo, Spain; 15 Department of Child and Adolescent Psychiatry, Institute of Psychiatry, Psychology & Neuroscience (IoPPN), King’s College London, London, UK; 16 Department of Legal Medicine, Psychiatry and Pathology, Facultad de Medicina, Universidad Complutense de Madrid (UCM), Madrid, Spain; 17 Department of Psychiatry, Instituto de Investigación Sanitaria Hospital 12 de Octubre (imas12), Madrid, Spain; 18 Department of Psychiatry, Hospital Universitari Institut Pere Mata, Reus, Spain; 19 Neurociències i Salut Mental, Institut d’Investigació Sanitària Pere Virgili-CERCA, Reus, Spain; 20 Department Medicina i Cirurgia, Universitat Rovira i Virgili, Tarragona, Spain; 21 Psychosocial Rehabilitation Unit, Hospitalarias Arturo Soria Foundation, Madrid, Spain; 22 BIORABA, Department of Psychiatry, Hospital Universitario de Álava, CIBERSAM, UPV/EHU, Vitoria, Spain; 23 Institut de Salut Mental, Hospital del Mar, Barcelona, Spain; 24 Mental Health Research Group, Hospital del Mar Research Institute, CIBERSAM, Barcelona, Spain; 25 Department of Psychiatry, Zucker School of Medicine at Hofstra/Northwell, Hempstead, NY, USA; 26 Research Group on Personal Autonomy, Dependency and Severe Mental Disorders, INCLIVA Biomedical Research Institute, Valencia, Spain; 27 Department of Developmental and Educational Psychology, Faculty of Psychology and Speech and Language Therapy, University of Valencia, Valencia, Spain; 28 Teaching Unit of Psychiatry and Psychological Medicine, Department of Medicine, University of Valencia, Valencia, Spain; 29 Department of Psychiatry, Hospital de la Santa Creu i Sant Pau, Institut de Recerca Sant Pau (IR SANT PAU), Barcelona, Spain; 30 Department of Psychiatry, Mental Health and Addictions, Hospital Universitari Santa Maria, Lleida, Spain; 31 Biological Foundations of Mental Disorders Group, Institut de Recerca Biomèdica (IRB) de Lleida, Lleida, Spain; 32 Department of Psychiatry, School of Medicine, University of Seville, Seville, Spain; 33 Translational Psychiatry Group, Seville Biomedical Research Institute (IBiS)-CSIC, Seville, Spain; 34 Department of Psychiatry, Hospital Álvaro Cunqueiro, Servicio Galego de Saúde (SERGAS), Vigo, Spain; 35 Translational Neuroscience Group, Galicia Sur Health Research Institute (IIS Galicia Sur), SERGAS-UVIGO, Vigo, Spain; 36 Department of Psychiatry, HGU Gregorio Marañón, Madrid, Spain; 37 Department of Psychiatry, Hospital Dr. Rodríguez Lafora, Madrid, Spain; 38 Department of Psychiatry, Universidad Francisco de Vitoria, Madrid, Spain; 39 Mental Health Group, Consorci Corporació Sanitària Parc Taulí – I3PT – CIBERSAM, Sabadell, Barcelona, Spain; 40 Departament de Psiquiatria i Medicina Legal, Universitat Autònoma de Barcelona, Barcelona, Spain; 41 Department of Psychiatry, University Hospital Virgen del Rocio, Andalusian Health Service, Seville, Spain; 42 Translational Psychiatry Group, Seville Biomedical Research, Seville, Spain; 43 Institute (IBiS)-CSIC, Foundation for Health Research Management in Seville, Seville, Spain; 44 Department of Psychiatry, Hospital Clínic Universitari, Valencia, Spain

**Keywords:** first-episode psychosis, functional capacity, performance-based assessment, schizophrenia, validation, VRFCAT

## Abstract

**Background:**

The Virtual Reality Functional Capacity Assessment Tool (VRFCAT) is a performance-based measure developed to assess functional capacity through simulations of daily activities. This study examined its psychometric properties in a Spanish sample, including individuals with first-episode psychosis (FEP), schizophrenia, and healthy controls.

**Methods:**

A total of 370 participants (99 FEP, 116 schizophrenia, and 155 controls) completed the VRFCAT in a multicenter study. Internal consistency (McDonald’s omega), discriminative validity (group comparisons and ROC curves), and convergent validity via correlations with cognitive performance and clinical symptoms were examined. Reference percentiles were calculated from the healthy control sample using quantile regression, stratified by age and education.

**Results:**

Item-level VRFCAT completion times showed acceptable to good internal consistency overall and in controls and schizophrenia samples, but poor in FEP. Differences in VRFCAT performance emerged (*χ* ^2^ = 108.88, *p* < .001), with controls performing best, schizophrenia worst, and FEP in between. ROC analyses indicated good discriminative accuracy in distinguishing patients from controls (area under the curve [AUC] = 0.779, sensitivity = 80.0%, specificity = 64.2%); but limited discrimination between schizophrenia and FEP. Age and education, but not sex, significantly affected performance. VRFCAT showed small-to-moderate correlations with cognitive performance, and no significant associations with symptom severity.

**Conclusions:**

The VRFCAT is a reliable and valid tool for assessing functional capacity in Spanish-speaking individuals with psychotic disorders. Its ecological validity, objectivity, psychometric properties, brief administration time, and ease of use support its potential use in clinical and research settings for evaluating functional recovery and treatment outcomes.

## Introduction

Functional impairment is a core feature of psychotic disorders and a major determinant of quality of life [1]. Growing interest has focused on the assessment of functional capacity, defined as the ability to carry out everyday tasks under standardized conditions, as a distinct and valid construct that provides a direct and ecologically meaningful estimate of disability [2]. Functional capacity is more proximal to cognitive functioning than real-world behavior, and serves as a valuable intermediate outcome in clinical trials, particularly for assessing response to interventions before changes are observed in actual functioning [3–5]. Compared to traditional assessments based on self-report or informant ratings, performance-based measures offer a more ecologically valid and objective means of evaluating an individual’s functional potential, while minimizing the influence of insight, environment, or support systems [6]. These tools have become increasingly central in schizophrenia research, particularly in efforts to define and measure functional recovery, where they are often used as co-primary outcomes to evaluate the impact of cognitive interventions.

The Virtual Reality Functional Capacity Assessment Tool (VRFCAT) is a computerized measure of functional capacity through realistic simulations of everyday activities [7]. Unlike traditional role-play tasks or paper-based instruments, the VRFCAT presents a series of interactive scenarios – such as meal preparation, using transportation, shopping, and handling money – designed to capture essential skills for independent living in a dynamic and ecologically valid environment. Its digital format enhances standardization, reduces administrator burden, and minimizes variability across sites, making it particularly suitable for research and clinical purposes. Importantly, the VRFCAT has demonstrated strong psychometric properties, including good test–retest reliability, sensitivity to cognitive and functional deficits, and convergent validity with both cognitive performance and real-world outcomes [7, 8]. However, key limitations remain: internal consistency – critical for interpreting the global score – has not been formally reported, and no validation studies or reference values based on healthy control samples are currently available for Spanish-speaking populations.

This study aimed to validate the VRFCAT in a Spanish sample of individuals with psychosis (schizophrenia and first-episode psychosis, FEP) and healthy controls by: (i) examining internal consistency; (ii) assessing discriminative and convergent validity; and (iii) providing reference percentiles from the healthy control sample, stratified by sex, age, and education, to support clinical use. Based on previous literature, we hypothesized that the VRFCAT would demonstrate acceptable internal consistency and validity in a Spanish sample. Specifically, we expected patients with psychosis (schizophrenia and FEP) to perform significantly worse than healthy controls, and VRFCAT performance to show moderate associations with cognitive functioning but minimal or no associations with symptom severity.

## Method

### Participants and procedure

Participants were recruited as part of a multicenter study conducted in 15 public mental health centers and university hospitals across Spain, aimed at validating digital tools for the assessment of cognitive functioning and everyday functioning in individuals with psychotic disorders (Project Code: PI20/00066). The final sample included individuals diagnosed with chronic schizophrenia (hereafter referred to as schizophrenia) or FEP, identified through clinician referral based on convenience sampling, and healthy controls selected to achieve a comparable distribution of age, sex, and educational level.

A total of 403 participants were initially recruited, including 117 individuals with FEP, 125 with schizophrenia, and 161 healthy controls. After excluding participants with missing VRFCAT data, the final sample included 370 individuals: 99 with FEP, 116 with schizophrenia, and 155 controls.

Participants were assigned to one of three groups: FEP, schizophrenia, or controls. Patients were eligible if they: (1) were aged 18–60 years, (2) had a DSM-5 diagnosis of a schizophrenia-spectrum psychotic disorder, (3) had adequate Spanish proficiency, and (4) could provide informed consent. The exclusion criteria were as follows: (1) organic brain pathology or neurological illness, (2) intellectual disability (DSM-5), and (3) current or recent (past 6 months) substance dependence, assessed via the Comprehensive Assessment of Symptoms and History [CASH; 9]. Patients were classified as FEP if they had initiated antipsychotic treatment within the past three years; otherwise, they were classified as having chronic schizophrenia.

Healthy controls met the same age and language criteria and were required to provide informed consent. Exclusion criteria included any past or current mental or neurological disorder, intellectual disability, substance use disorder, or psychotropic medication use.

Data were collected between July 2022 and December 2024. Trained research staff administered the assessments in a single session following standardized instructions. The full protocol lasted approximately 90 minutes. Written informed consent was obtained from all participants prior to the evaluation, and the study received ethical approval from the ethics committee of the principal center (code: PI20/00066) and from the corresponding ethics committees at each participating site.

### Measures

#### Virtual Reality Functional Capacity Assessment Tool [VRFCAT]

The VRFCAT is a computerized performance-based measure developed to assess functional capacity by simulating everyday tasks in a realistic virtual environment (Keefe et al., 2016). The tool presents participants with a sequence of 12 objectives embedded in a storyboard format that reflects four core domains of daily functioning: meal planning, transportation use, shopping, and money management. The Spanish version of the VRFCAT was administered on a tablet with interactive first-person navigation. The VRFCAT provides three main outcome measures: total time to completion, total number of errors, and number of forced progressions. Forced progressions occur when a participant is unable to complete a task within the maximum time limit of 300 s, in which case the system automatically registers the maximum time and advances to the next objective. The original validation study of the VRFCAT [7] found strong overlap between total time to completion and total number of errors (*r* ≈ .70). Given its greater sensitivity and reduced ceiling effects compared to total number of errors, total time to completion was selected as the primary outcome measure. A higher total time to completion indicates poorer functional capacity.

#### Brief Assessment of Cognition – App version [BAC App]

The BAC App [10] is a tablet-based version of the Brief Assessment of Cognition in Schizophrenia [BACS; 11], designed to assess six cognitive domains relevant to clinical populations: episodic memory, working memory, verbal fluency, processing speed, executive functioning, and psychomotor speed. The full battery takes approximately 30 minutes to complete. In the current study, we used raw scores from the BAC App. A recent validation using the same Spanish-speaking sample as the present study reported good internal consistency (*α* = .76–.87), moderate positive correlations between the BAC App composite score and estimated IQ (up to *r* = .49), and good discriminative validity in distinguishing clinical from non-clinical participants [12].

#### Positive and Negative Syndrome Scale [PANSS]

The PANSS includes three subscales: Positive Symptoms (seven items), Negative Symptoms (seven items), and General Psychopathology (16 items). In the present study, we used the validated Spanish version of the PANSS [13, 14].

#### Vocabulary subtest of the Wechsler Adult Intelligence Scale – Third Edition [WAIS-III]

The Spanish version Vocabulary subtest from the WAIS-III was used to estimate the premorbid intelligence quotient (IQ). Raw scores were converted to age-adjusted scaled scores using normative data from the WAIS-III manual [15].

### Sociodemographic and clinical variables

Sociodemographic data, including age, sex, ethnicity, educational level, and current employment status, were collected. Clinical history was obtained through a structured interview that included mental diagnosis based on DSM-5 criteria, age of onset of duration of untreated illness (DUI), and duration of untreated psychosis (DUP).

### Data analysis

Descriptive statistics for each VRFCAT item and the total score, including means, medians, standard deviations, interquartile ranges (IQR), skewness, and kurtosis, were performed separately for individuals with FEP, schizophrenia, and healthy controls. Due to the non-normal distribution of VRFCAT data, nonparametric tests were used. The internal consistency of the VRFCAT was assessed using McDonald’s omega coefficient (*ω*), which is more appropriate for ordinal data and heterogeneous item loadings. To examine the impact of sociodemographic variables (sex, age, and years of education) on VRFCAT performance, we conducted Mann–Whitney *U* tests and Spearman’s rank-order correlations within each group.

Quantile regression analyses were conducted on the healthy control sample to generate reference percentiles (10th, 25th, 50th, 75th, and 90th) for VRFCAT total time to completion, stratified by age and educational level. Age was categorized into three groups: 18–29, 30–44, and 45 years or older; education was grouped into 5–9, 10–14, and 15 years or more of formal schooling. These categories were used to ensure robust percentile estimation and preserve clinical interpretability. Between-group comparisons (FEP, schizophrenia, and healthy controls) were conducted using Kruskal–Wallis tests, followed by Dunn’s post hoc pairwise comparisons with *p*-values adjusted using Holm’s correction for multiple testing. Discriminative validity was assessed using Receiver Operating Characteristic (ROC) curve analyses, reporting the area under the curve (AUC), sensitivity, specificity, and the Youden Index, which reflects the maximum combined value of sensitivity and specificity.

Finally, convergent validity was examined using Spearman’s rank-order correlations between VRFCAT total time to completion and cognitive performance (BAC App) as well as estimated premorbid IQ (WAIS-IV Vocabulary), across the three groups. Associations with clinical symptom severity (PANSS) were also explored to examine discriminant validity.

All statistical tests were two-tailed, with the significance threshold set at *p* < .05. Analyses were conducted using IBM SPSS Statistics (version 26) and JASP (version 0.19.3).

## Results

### Sociodemographic and clinical characteristics of the sample

Table 1 shows demographic and clinical characteristics across groups. Significant differences were found across groups in age, years of education, ethnicity, marital status, and employment status. In contrast, sex distribution did not differ significantly between groups.

**Table 1.** Demographic and clinical characteristics of FEP, schizophrenia, and healthy controls

	FEP(*n* = 99)	Schizophrenia (*n* = 116)	HC(*n* = 155)	Statistic	*P*-value
Age, Md (IQR)	24 (20.5–33)	40 (31–48)	31 (26–37)	*H* = 67.930	< .001
Sex, *n* (%)				*χ* ^2^ = 3.068	.216
Male	62 (62.6)	77 (66.4)	87 (56.1)		
Female	37 (37.4)	39 (33.6)	68 (43.9)		
Years of education, Md (IQR)	14.0 (11–17)	12.0 (10–16)	16 (12–19)	*H* = 22.343	< .001
Marital status, *n* (%)				*χ* ^2^ = 23.047	< .001
Single	81 (81.8)	94 (81.0)	92 (59.4)		
Married	12 (12.1%)	13 (11.2)	47 (30.3)		
Other	5 (5.1)	9 (7.8)	12 (7.7)		
Ethnicity, *n* (%)				*χ* ^2^ = 30.657	< .001
Caucasian	52 (52.5)	94 (81.0)	126 (81.3)		
Latino	43 (43.4)	20 (17.2)	27 (17.4)		
Other	4 (4.0)	2 (1.7)	2 (1.3%)		
Employment status, *n* (%)				χ^2^ = 165.437	< .001
Student	26 (26.3)	11 (9.5)	27 (17.4)		
Employed	36 (36.4)	21 (18.1)	112 (72.3)		
Unemployed	20 (20.2)	19 (16.4)	9 (5.8)		
Disability	2 (2.0)	48 (41.4)	2 (1.3)		
Other	13 (13.1)	17 (14.7)	5 (3.2)		
DUP, Md (IQR)	2 (1–7)	2 (1–24)	–	*U* = 4814.0	.078
DUI, Md (IQR)	10 (2–24)	12.0 (1–95)	–	*U* = 5099.0	.435
PANSS positive, Md (IQR)	10 (7–18)	11.0 (8–16)	–	*U* = 5244.0	.893
PANSS negative, Md (IQR)	11 (7–17)	15.0 (10–20)	–	*U* = 3905.5	.008
PANSS general, Md (IQR)	25.5 (20–35.5)	25.0 (20–31)	–	*U* = 4922.5	.695

Abbreviations: DUP, duration of untreated psychosis (in months); DUI, duration of untreated illness (in months); FEP, first-episode psychosis; HC, healthy controls; IQR, interquartile range (25th–75th percentile); Md, median; PANSS, Positive and Negative Syndrome Scale; H, Kruskal–Wallis test statistic; *U*, Mann–Whitney *U* test statistic.

We compared sex, age, and education between the clinical (*n* = 215) and control (*n* = 155) groups to assess demographic comparability. No significant differences were found in sex (*χ* ^2^(1) = 2.75, *p* = .097) or age (*U* = 15,455.5, *p* = .628). Although years of education differed significantly (*U* = 12,257.5, *p* < .001), the nonparametric effect size was modest (*r* = .23).

Table 2 presents descriptive statistics for each VRFCAT item and the total time to completion across the three groups. The distribution showed marked deviations from normality, with several items exhibiting substantial positive skewness and kurtosis.

**Table 2.** Descriptive statistics for VRFCAT items in FEP, schizophrenia, and healthy control groups

VRFCAT item	Mean	SD	Median	IQR	Skewness	Kurtosis
*FEP*						
1	49.9	16.5	46.3	48.1–45.7	5.9	42.2
2	87.7	41.3	74.8	100.0–63.9	3.1	13.0
3	121.6	77.3	104.0	168.1–58.0	0.8	−0.2
4	22.8	7.0	21.2	22.0–21.0	7.8	69.4
5	12.7	6.0	10.7	13.1–9.8	3.8	17.7
6	69.3	24.7	65.6	67.2–65.0	8.4	80.0
7	44.3	11.5	41.6	46.1–38.0	1.9	5.7
8	27.4	20.5	23.0	27.7–19.0	6.2	45.5
9	134.8	49.5	128.0	162.0–102.0	0.9	1.7
10	85.8	54.0	66.0	100.7–49.9	1.9	4.3
11	68.2	16.5	64.5	66.1–64.0	5.2	42.0
12	45.4	39.8	34.0	51.0–24.8	3.7	18.1
**Total time**	769.8	193.5	722.7	855.0–629.7	0.6	1.2
*Schizophrenia*						
1	51.1	8.6	47.2	52.1–46.2	2.3	5.4
2	101.8	43.2	91.1	120.5–68.8	1.9	4.6
3	148.7	89.8	125.9	217.0–71.9	0.5	−1.1
4	26.3	22.0	21.7	22.5–21.0	7.3	59.5
5	18.4	16.7	12.1	18.0–10.6	3.8	17.5
6	79.4	42.6	66.2	73.2–65.2	4.4	19.3
7	63.1	44.2	49.2	67.8–39.3	3.6	15.5
8	31.8	12.4	29.6	38.0–23.2	1.6	3.1
9	194.8	70.4	183.8	269.3–133.9	0.3	−1.3
10	108.1	65.5	86.4	125.4–61.5	1.5	1.8
11	77.8	39.6	65.0	68.4–64.0	3.8	14.1
12	74.1	59.9	52.6	90.0–33.0	2.0	4.3
**Total time**	975.5	300.2	943.6	1088.1–737.8	1.2	1.9
*Controls*						
1	50.5	19.5	46.1	45.6–48.0	6.5	46.3
2	78.8	26.4	69.6	60.1–93.9	1.5	3.2
3	72.7	46.0	50.0	39.2–99.3	1.9	5.0
4	23.6	6.7	21.3	21.0–22.6	3.9	16.9
5	14.0	10.2	10.7	9.8–13.4	5.1	32.7
6	68.0	7.4	65.7	65.0–67.0	3.7	15.3
7	44.6	14.3	41.0	36.9–47.2	3.3	16.4
8	25.1	7.5	23.6	19.6–28.4	1.7	4.3
9	116.7	41.3	108.1	91.0–128.1	1.9	5.2
10	68.3	40.7	56.0	47.3–72.5	3.4	14.1
11	67.9	20.2	64.8	64.0–66.1	10.4	116.2
12	37.1	30.0	29.8	21.1–43.2	5.1	39.0
**Total time**	667.2	149.0	637.0	575.5–714.7	2.5	10.3

Abbreviations: FEP, First-Episode Psychosis; IQR, Interquartile Range (25th to 75th percentile); SD, standard deviation; VRFCAT, Virtual Reality Functional Capacity Assessment Tool.

### Internal consistency

Internal consistency was acceptable to good across groups: for the total sample, *ω* = 0.80 (95% CI [0.77–0.83]); for the clinical group, *ω* = 0.76 (95% CI [0.71–0.81]); and for controls, *ω* = 0.71 (95% CI [0.64–0.78]). When FEP and schizophrenia groups were examined separately, internal consistency was acceptable in the schizophrenia (*ω* = 0.77, 95% CI [0.71–0.83]), but low in FEP (*ω* = 0.34, 95% CI [0.20–0.49]).

### Effects of sex, age, and education on VRFCAT performance

No sex differences in VRFCAT performance were observed within any group (all *p* > 0.05). In contrast, performance was significantly associated with age and education in some groups. In the FEP group, neither age nor education showed significant associations with VRFCAT performance (age: *ρ* = .165, *p* = .106; education: *ρ* = −.167, *p* = .099). In the schizophrenia group, older age was moderately correlated with slower VRFCAT performance (*ρ* = .408, *p* < .001), and fewer years of education were associated with poorer performance (*ρ* = −.221, *p* = .017). When combining the FEP and schizophrenia groups, age and education were significantly associated with VRFCAT total score (age: *ρ* = .403, *p* < .001; education: *ρ* = −.243, *p* < .001). In healthy controls, associations were weaker: VRFCAT showed a modest negative correlation with years of education (*ρ* = −.158, *p* = .050), and a positive but non-significant correlation with age (*ρ* = .150, *p* = .067). Based on these findings, percentile reference values were derived from the healthy control group according to age and education, but not by sex.

### Reference values from the healthy control sample

Reference values for VRFCAT total time to completion were calculated using quantile regression in the control sample, stratified by age group and educational level. Percentiles (10th, 25th, 50th, 75th, and 90th) were generated to enhance the clinical utility and interpretability of the measure (Table 3).

**Table 3.** Estimated VRFCAT reference values by age and education level derived from healthy controls

	Years of education			
Age group (years)	5–9	10–14	15+	Percentile
18–29	537	511	512	90
	682	597	583	75
	780	625	611	50
	809	674	672	25
	886	736	754	10
30–44	555	529	530	90
	660	575	561	75
	798	642	628	50
	845	710	708	25
	924	775	792	10
45+	570	543	544	90
	666	582	567	75
	846	690	677	50
	909	774	772	25
	978	828	845	10

*Note*: Values represent VRFCAT total time to completion (in seconds) estimated from quantile regression according to age group and education level. Percentiles (10th, 25th, 50th, 75th, 90th) are presented in descending order to reflect that lower rows correspond to lower performance, as higher completion times indicate poorer functional capacity. For example, a 35-year-old control participant with 12 years of education who takes 775 seconds to complete the VRFCAT would fall at the 10th percentile for their demographic group, suggesting a performance approximately one standard deviation below the expectations derived from the healthy control sample.

### Discriminative validity

ROC analyses were conducted to assess the ability of the VRFCAT total time to completion to distinguish between groups. The AUC for differentiating the clinical group (schizophrenia and FEP combined) from healthy controls was 0.779 (95% CI [0.732, 0.826]), indicating good accuracy. The optimal cutoff point (732 s) was identified based on the Youden Index (0.44), which yielded a sensitivity of 80.0% and specificity of 64.2%. When comparing schizophrenia and FEP, the AUC was 0.721 (95% CI [0.653, 0.788]), indicating modest discriminative power. The optimal cutoff point (746 s), based on the Youden Index (0.28), yielded a sensitivity of 74.1%, specificity of 53.5%.

### Group differences in item-level performance

Table 4 presents group differences in VRFCAT. Significant effects were found for all items and for the total time to completion (all *p* < .05). *Post hoc* comparisons revealed that participants with schizophrenia consistently had the longest completion times, performing significantly worse than both healthy controls and individuals with FEP on all items except item 4, for which no significant differences were found between schizophrenia and controls. This pattern was further confirmed by the total score, which also differed significantly across groups (χ^2^ = 108.9, *p* < .001): the schizophrenia group showed the poorest performance, controls the best, and the FEP group fell in between.

**Table 4.** Kruskal–Wallis test comparisons of VRFCAT item-level and total time to completion across FEP, schizophrenia, and control groups

			Mean ranks			
Item	*χ* ^2^(df = 2)	*p*-value	FEP(*n* = 99)	SCZ(*n* = 116)	HC(*n* = 155)	Post hoc (Dunn)^a^
*1. Pick up the recipe*	22.83	< .001	173.69	224.38	163.95	SCZ > FEP (*z* = −3.464, *p* = .001) SCZ > HC (*z* = 4.603, *p* < .001)
*2. Search for Ingredients*	29.03	< .001	182.62	226.94	156.32	SCZ > FEP (*z* = −3.029, *p* = .005) SCZ > HC (*z* = 5.378, *p* < .001)
*3. Cross off correct ingredients and pick up the bus schedule*	68.19	< .001	206.49	237.42	133.23	SCZ > HC (*z* = 7.936, *p* < .001) FEP > HC (*z* = 5.325, *p* < .001) SCZ > FEP (*z* = −2.114, *p* = .035)
*4. Pick up the billfold*	9.099	0.011	164.3	207.84	182.32	SCZ > FEP (*z* = −2.977, *p* = .009)
*5. Exit the apartment*	21.76	< .001	165.21	223.78	169.82	SCZ > HC (*z* = 4.110, *p* < .001) SCZ > FEP (*z* = −4.003, *p* < .001)
*6. Get on the bus to the grocery store*	13.02		165.84	214.51	176.35	SCZ > FEP (*z* = −3.327, *p* = .003) SCZ > HC (*z* = 2.908, *p* = .007)
*7. Pay for the bus*	31.27	< .001	167.86	231.4	162.42	SCZ > HC (*z* = 5.253, *p* < .001) SCZ > FEP (*z* = −4.342, *p* < .001)
*8. Select an aisle*	34.08	< .001	160.48	233.43	165.61	SCZ > HC (*z* = 5.165, *p* < .001) SCZ > FEP (*z* = −4.985, *p* < .001)
*9. Shop for groceries*	100.48	< .001	177.92	263.28	132.13	SCZ > HC (*z* = 9.990, *p* < .001) SCZ > FEP (*z* = −5.834, *p* < .001) FEP > HC (*z* = 3.328, *p* < .001)
*10. Pay for groceries*	47.30	0.013	188.09	236.16	145.93	SCZ > HC (*z* = 6.872, *p* < .001) SCZ > FEP (*z* = −3.285, *p* = .002) FEP > HC (*z* = 3.064, *p* = .002)
*11. Get on the bus to go home*	8.69	< .001	169.46	209.2	178.01	SCZ > FEP (*z* = −2.717, *p* = .020) SCZ > HC (*z* = 2.377, *p* = .035)
*12. Pay for the bus*	54.96	< .001	175.11	244.32	148.11	SCZ > HC (*z* = 7.327, *p* < .001) SCZ > FEP (*z* = −4.729, *p* < .001) FEP > HC (*z* = 1.962, *p* = .050)
**Total time**	108.88	< .001	189.24	262.44	125.54	SCZ > HC (*z* = 10.427, *p* < .001) SCZ > FEP (*z* = −5.002, *p* < .001) FEP > HC (*z* = 4.630, *p* < .001)

Abbreviations: Df, degrees of freedom; FEP, first-episode psychosis; HC, healthy controls; SCZ, schizophrenia.

a Post hoc *p*-values are adjusted using the Holm correction for multiple comparisons. Group comparisons (e.g., SCZ > FEP) reflect differences in mean ranks; higher ranks correspond to longer completion times and thus poorer VRFCAT performance.

### Associations with cognitive performance, IQ, and clinical symptoms

As shown in Table 5, VRFCAT total time to completion was significantly correlated with several cognitive measures across all groups, with small to moderate associations, strongest in the schizophrenia group. No significant association was observed with symptom severity in either clinical group.

**Table 5.** Spearman correlations between VRFCAT total time to completion and IQ, BAC App, and PANSS scores in individuals with FEP, schizophrenia, and healthy controls

	FEP(*n* = 99)		Schizophrenia(*n* = 116)		Healthy Controls(*n* = 155)	
Variable	ρ	*p*-value	ρ	*p*-value	ρ	*p*-value
IQ (Vocabulary)	0.001	0.994	**−0.306**	0.001	**−0.174**	0.037
BAC App – verbal memory	**−0.352**	< 0.001	**−0.387**	< 0.001	**−0.172**	0.033
BAC App – digit sequencing	−0.127	0.209	**−0.310**	0.001	**−0.185**	0.021
BAC App – token motor task	**−0.426**	< 0.001	**−0.498**	< 0.001	**−0.225**	0.005
BAC App – fluency	−0.065	0.521	**−0.247**	0.008	**−0.214**	0.007
BAC App – symbol coding	**−0.368**	< 0.001	**−0.622**	< 0.001	**−0.301**	< 0.001
BAC App – tower of London	**−0.218**	0.030	**−0.572**	< 0.001	−0.110	0.172
PANSS positive	0.092	0.379	0.102	0.281	–	–
PANSS negative	0.128	0.230	0.077	0.419	–	–
PANSS general	0.082	0.441	0.077	0.418	–	–

Abbreviations: BAC App, Brief Assessment of Cognition App; FEP, First-Episode Psychosis; IQ, estimated Intelligence Quotient; PANSS, Positive and Negative Syndrome Scale; VRFCAT, Virtual Reality Functional Capacity Assessment Tool.

*Note:* Bolded values indicate statistically significant correlations (p < .05).

## Discussion

The aim of the present study was to examine the psychometric properties of the VRFCAT in a Spanish sample of individuals with FEP and schizophrenia and controls and to provide reference values from the control group to support the interpretation of performance in individuals with psychosis. First, item-level completion times on the VRFCAT demonstrated acceptable to good internal consistency across the total sample and groups. Second, the tool showed adequate discriminative validity, effectively distinguishing between groups, with controls performing best, schizophrenia the worst, and FEP at an intermediate level. ROC curve analyses further supported the discriminative ability of the VRFCAT, revealing good accuracy in differentiating the clinical group from healthy controls, but only modest performance in distinguishing between FEP and schizophrenia. Third, convergent validity was supported by modest correlations between VRFCAT performance and cognitive functioning. In contrast, no significant associations were found with symptom severity, consistent with the expected discriminant validity. Finally, we provided stratified reference percentiles from the healthy control sample by age and education to aid interpretation of scores in clinical and research contexts.

The current findings are broadly consistent with those of previous studies, validating the VRFCAT as a sensitive tool for assessing functional capacity in individuals with psychosis. Across several studies, the VRFCAT has consistently demonstrated sensitivity to group differences, with individuals with schizophrenia or FEP performing significantly worse than healthy controls [7, 8, 16, 17]. Importantly, its ability to differentiate between individuals with schizophrenia and those with FEP was modest. This may reflect the early emergence of functional deficits and heterogeneity both within and across phases of psychosis, as well as the nature of the VRFCAT as a measure of potential functional ability under optimal conditions, which may not fully capture the real-world functional deterioration typically observed in chronic stages.

In terms of reliability, the test–retest reliability of the VRFCAT has been previously demonstrated in both clinical and non-clinical populations [7, 18]. However, to our knowledge, internal consistency has not been assessed to date. In our study, we observed acceptable to good reliability across groups, as indicated by McDonald’s omega coefficients, supporting the robustness and reliability of the total score as a performance-based outcome. However, reliability was notably lower in the FEP subgroup, suggesting that measurement consistency may be affected in the early stages of illness, possibly due to greater clinical heterogeneity, and highlighting the need for further investigation in this population.

Although few studies have examined the influence of sociodemographic variables on VRFCAT performance [7, 8, 18, 19]. Our findings are consistent with the limited evidence available. Older participants and those with lower educational attainment had slower completion times. This pattern aligns with the findings of Atkins et al. [18], who used the VRFCAT in a healthy sample and reported significant age-related differences, likely reflecting declines in processing speed, working memory, and executive functioning, which impact the performance of complex daily tasks. Additionally, Ventura et al. [8] found that while patient–control differences in VRFCAT total time to completion remained significant after adjusting for parental education (used as a proxy for expected educational level), the effect was attenuated, supporting the role of educational background in influencing functional capacity performance, likely through its impact on the cognitive and practical skills needed for everyday tasks assessed by the VRFCAT. In contrast, no significant sex differences were found in our sample, neither in patients nor in controls, a finding that is in line with previous studies using this tool [7, 8, 19].

Slower VRFCAT performance was significantly associated with poorer cognitive functioning. However, the small to moderate effect sizes of these correlations suggest that these constructs are related but distinct. Correlations with neurocognitive performance assessed using the MATRICS Consensus Cognitive Battery (MCCB) have been consistently reported in previous studies [7, 8, 17, 20]. Harvey et al. [20] conducted a joint factor analysis of cognitive and functional capacity measures and found that, while the relationship between the MCCB and the UCSD Performance-Based Skills Assessment (UPSA) – a widely used measure of functional capacity – fit a one-factor model, whereas the VRFCAT required a two-factor solution, with the factors being moderately correlated. These findings support the notion that the VRFCAT captures functionally relevant abilities that extend beyond those measured by traditional neurocognitive assessments. Our results should also be considered in light of current international recommendations, such as the recent European Psychiatric Association guidance on the treatment of cognitive impairment in schizophrenia, which emphasizes cognition as a key therapeutic target to improve functional capacity and, ultimately, daily functioning [21].

This study has several limitations. First, the cross-sectional design precludes any conclusions about the temporal stability of VRFCAT performance or its predictive value for real-world functional outcomes. Second, convergent validity was examined only through associations with few cognitive measures (BAC App and IQ estimation), and no additional functional capacity or real-world functioning assessments were included for comparison. In particular, the lack of direct comparison with other validated performance-based measures represents a key limitation for assessing convergent validity; this should be addressed in future studies through concurrent administration of the VRFCAT and the UPSA-Brief [22] or similar instruments. Third, participants were recruited through convenience sampling. Fourth, internal consistency for the VRFCAT in the FEP subgroup was unacceptably low, raising concerns about the reliability of the measure in early psychosis. Finally, the sample size was determined based on psychometric considerations derived from the Rasch model [23] rather than on a conventional a priori power analysis. Although the final sample (215 patients and 155 controls) did not reach the originally intended size (≈250 per group) recommended for maximum calibration stability, it was sufficient for the planned validation analyses.

These findings lead to several recommendations for future research using the VRFCAT. First, given the non-normal distribution typically observed in task completion times, non-parametric statistical methods are strongly recommended, especially with small samples (*N* ≤ 30) or strong deviations from normality. Second, given that total time to completion is frequently used as the primary outcome in VRFCAT studies, it is surprising that its internal consistency has not been previously reported; this metric should be routinely included in future research. Third, participant age and educational attainment should be considered in both study design and interpretation of results, as these variables have consistently shown to influence VRFCAT performance. Additionally, although no significant sex differences have been observed to date, continued evaluation of potential gender-related effects is warranted to ensure that conclusions drawn from VRFCAT data are generalizable to both men and women.

The present study has several strengths worth noting. First, it provides the first validation of the VRFCAT in a Spanish-speaking sample, addressing an important gap in the international literature and expanding the potential for cross-cultural research and clinical application. Moreover, the study was conducted across multiple centers, enhancing the generalizability of the findings within the Spanish clinical context. Interestingly, previous international research has shown that regional differences in performance on functional capacity assessments such as the VRFCAT are modest compared to the robust and consistent differences observed between individuals with schizophrenia and healthy controls [16]. This supports that, despite potential cultural and geographical variations, the pattern of functional impairment captured by the VRFCAT is likely to be generalizable across settings, supporting its utility for both clinical and research purposes beyond the Spanish population. Second, the inclusion of individuals with schizophrenia, FEP, and demographically comparable controls allows for a more detailed understanding of functional capacity across different stages of illness. Third, the use of quantile regression to generate stratified reference values from the healthy control sample by age and education enhances the clinical interpretability of individual scores.

In summary, the present findings support the VRFCAT as a reliable and valid tool for assessing functional capacity in individuals with psychosis within a Spanish-speaking population. The instrument demonstrated adequate psychometric properties, including internal consistency, discriminative ability, and convergent validity with cognitive functioning. Its performance-based nature, ecological validity, and standardized format make it particularly valuable for both research and clinical contexts, especially given its brief administration time, ease of use, and minimal demands on staff. The VRFCAT represents a promising measure for assessing functional recovery and may serve as a useful endpoint in intervention studies targeting everyday functioning in psychosis.

## Data Availability

The datasets generated and/or analyzed during the current study are available from the corresponding author on reasonable request.
